# Corticospinal Excitability During Explosive Voluntary Contractions and Its Association With Rapid Torque Production

**DOI:** 10.1111/ejn.70321

**Published:** 2025-11-18

**Authors:** Federico Castelli, Omar S. Mian, Adam Bruton, Ashika C. Valappil, Ricci Hannah, Neale A. Tillin

**Affiliations:** ^1^ School of Life and Health Sciences, Whitelands College University of Roehampton London UK; ^2^ Centre for Physical Activity, Health and Disease, Division of Sport Health and Exercise Sciences Brunel University London Uxbridge UK; ^3^ Centre for Human & Applied Physiological Sciences King's College London London UK

**Keywords:** corticospinal excitability, corticospinal inhibition, explosive strength, motor‐evoked potential, muscle activation, silent period duration, transcranial magnetic stimulation

## Abstract

We investigated relationships between rapid torque and corticospinal excitability (denoted by motor‐evoked potential; MEP) and inhibition (denoted by silent period duration; SPD) during explosive voluntary contractions, as well as differences in MEP and SPD between different phases of explosive contractions and at maximum voluntary contraction (MVC) plateau. In 14 adults, and across multiple repeated trials, quadriceps muscle MEP and SPD were measured at the early, middle and late phases of knee‐extensor isometric explosive contractions, and at the MVC plateau, using transcranial magnetic stimulation (TMS). Torque at equivalent time points was also measured on trials without TMS. Using repeated measures correlation applied to early phase data from TMS trials, we found MEP and torque (measured just prior to MEP) were correlated across trials within participants (*r* = 0.43, *p* < 0.001). Using Spearman rho correlations to investigate correlations across participants for each phase, we found MEP (averaged across phases up to the phase of interest) and torque (measured on non‐TMS trials) to be significantly correlated for the middle phase only (rho = 0.73, *p* = 0.004). Linear mixed effects models were used to investigate the effect of phase (three explosive phases and MVC plateau) on MEP and SPD. Absolute MEP, MEP normalised to maximal M‐wave and SPD all increased across the phases of explosive contraction and up to MVC plateau (fixed effects of phase, *p* < 0.025). Our results suggest corticospinal excitability may be an important determinant of rapid torque. Further, corticospinal inhibition and excitability both increase throughout the rising torque‐time curve and up to MVC plateau.

## Graphical abstract

Explosive torque production is associated with corticospinal excitability. We observed that motor‐evoked potential (MEP) amplitude, elicited via TMS, correlates with early and middle phase torque output. MEP amplitude increases with contraction phase, while inhibition (silent period) remains low early, supporting rapid muscle activation.

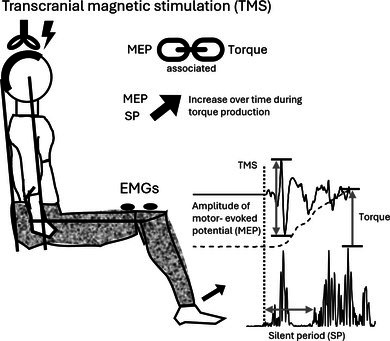

**Abbreviations**
AMT: active motor threshold
EMG: electromyography
GABA: gamma‐aminobutyric acid
MEP: motor‐evoked potential
MU: motor unit
MVC: maximal voluntary contraction
RF: rectus femoris
RmCorr: repeated measures correlation
RMS: root mean squared
RMT: resting motor threshold
RTD_max_
: maximal rate of torque development
SD: standard deviation
TMS: transcranial magnetic stimulation
VL: vastus lateralis
VM: vastus medialis

## 1 Introduction

The ability of muscles to rapidly increase joint torque is important for movements where the time to develop torque is limited, such as sprinting (Tillin et al. 2013) or balance recovery (Sundstrup et al. 2010). Rapid torque is often assessed by measuring torque at discrete time points from torque onset. These time points are typically selected to fall within separate phases of the rising torque time curve: early phase, where the rate of rise is slow; middle phase, where the rate of rise is fastest; and late phase, where the torque begins to plateau (Maffiuletti et al. 2016). Maximum voluntary contraction (MVC) torque is an important determinant of late‐phase torque (Folland et al. 2014), while muscle activation, determined by the number of motor units recruited and their discharge rates, is an important limiting factor of early‐ and middle ‐phase rapid torque (Folland et al. 2014; Del Vecchio et al. 2019). Despite the relevance of muscle activation, corticospinal behaviour during explosive contractions and its association with rapid torque is not well established. Such factors could be explored using transcranial magnetic stimulation (TMS), superimposed during the different phases of explosive voluntary contractions. Specifically, superimposed TMS elicits motor‐evoked potentials (MEPs) and silent period durations immediately after the MEP, in the EMG signal of the target muscles, which are thought to reflect corticospinal excitability (Rossini et al. 2015) and inhibitory mechanisms (Säisänen et al. 2008; Yacyshyn et al. 2016), respectively.

Compared with contractions involving slow torque production, explosive (rapid) contractions are characterised by lower motor unit activation thresholds (Desmedt and Godaux 1977) and high motor unit discharge rates (Van Cutsem et al. 1998; Duchateau and Baudry 2014; Del Vecchio et al. 2019). This high muscle activation seems necessary to maximise rapid torque production, a theory further supported by observations of positive correlations between agonist EMG amplitude in the early phase (0–50 ms) of EMG activity—which commences prior to torque onset (Tillin et al. 2010)—and both early‐ and middle‐phase rapid torque (Folland et al. 2014). To achieve high muscle activation (and thus high rapid torques), it is conceivable corticospinal excitability will need to be high, given that corticospinal excitability increases with contraction intensity (Todd et al. 2003; Goodall et al. 2009; Weavil et al. 2015). We might, therefore, expect MEP amplitude to be positively correlated with early‐ and middle‐phase rapid torque both within a person across separate contractions and across participants.

In addition to high corticospinal excitability, low corticospinal inhibition during the early phase of EMG activity may be necessary to optimise rapid torque production. Cortical inhibition, measured by reduced short‐interval intracortical inhibition, appears to decrease in preparation for an explosive contraction immediately prior to EMG onset (Baudry and Duchateau 2021). Should low inhibitory conditions persist after the onset of EMG activity during an explosive contraction, and should this low inhibition be necessary to optimise rapid torque, we may expect the TMS‐induced silent period duration to be short and negatively correlated with early‐ and middle‐phase rapid torque.

Immediately after the initial activation of motor units at the start of an explosive contraction, the motor unit discharge rate drops from up to ~189 to < 50 Hz (Desmedt and Godaux 1977; Van Cutsem et al. 1998; Del Vecchio et al. 2019) and remains this low at the plateau of an MVC (Pucci et al. 2006). Additionally, EMG amplitude increases rapidly in the early phase of explosive contraction, reaching nearly 120% of the EMG observed at MVC plateau after 75 ms, before declining to stabilise at 100% of MVC (Tillin et al. 2018). These results collectively suggest greater muscle activation in early phases compared with later phases of explosive contractions and MVC plateau. This dynamic modulation of muscle activation may be achieved through either: (i) corticospinal excitability starting high and decreasing and/or (ii) corticospinal inhibition starting low and increasing, throughout the different phases of explosive contraction and up to MVC plateau. To the best of our knowledge, neither (i) nor (ii) has been investigated in explosive contractions, while a study of ballistic wrist movements, showed MEP amplitude increased prior to movement onset but declined after movement onset (Mackinnon and Rothwell 2000). Further, the increase in MEP amplitude preceded an increase in EMG amplitude, characterised by greater MEP/EMG in the early phases compared with later phases of contraction, suggesting corticospinal excitability increases disproportionately with muscle activation in the early phase, but changes proportionately with muscle activation at later phases of rapid contraction (Mackinnon and Rothwell 2000). It is unclear if this phenomenon observed for dynamic rapid wrist movements is present during the isometric explosive contractions used to assess rapid torque production.

This study has two main aims. The first aim investigates the relationships between rapid torque in the different phases of explosive contraction and both MEP amplitude and silent period duration in the same phases. These relationships will be explored between contractions within people and across participants. We hypothesise that MEP amplitudes in the early and middle phases will be positively correlated with rapid torque, while silent period durations will be negatively correlated. The second aim investigates the possible differences in MEP amplitude and silent period durations between different phases of explosive contraction (early, middle and late) and MVC plateau. We hypothesise that MEP amplitudes (absolute and relative to background EMG) will be greater and silent period durations shorter in the early phase of explosive contraction than in the other contraction phases.

## 2 Method

### 2.1 Participants

Fourteen participants, nine males (age: 31 ± 5 years; height: 178.7 ± 6.6 cm; and mass: 79.2 ± 4.7 kg) and five females (age: 29 ± 6 years; height: 165.5 ± 5.5 cm; and mass: 59.3 ± 6.2 kg) were recruited to take part in this study. All participants habitually performed 120–180 min of moderate to high‐intensity activity per week and were deemed recreationally active (McKay et al. 2022). Participants were also free from injury and disease (screened via a questionnaire adapted from Balady et al. 1998) and free from contraindications to TMS (screened via a questionnaire adapted from Rossi et al. 2011). Due to changes in endogenous hormones throughout the menstrual cycle potentially affecting neuromuscular responses to TMS (Ansdell et al. 2019), female participants undertook experimental trials exclusively during their self‐reported early to mid‐follicular phase (first 10 days from the first day of menstruation), when endogenous hormone concentration is low and relatively stable (de Jonge et al. 2019). The University of Roehampton ethics committee approved the study, and all participants provided written informed consent before participating.

### 2.2 Overview

We have previously published some of the data collected for this study in a separate manuscript with the distinct aim of investigating the reliability of our methods (Castelli et al. 2025). Participants visited the laboratory on three separate occasions and were asked to avoid strenuous exercise and alcohol consumption for 24 h before each visit. Each session lasted approximately 120–150 min, with consecutive sessions separated by 3–7 days. The first visit was a familiarisation session, and the second and third were measurement sessions. The measurement sessions were performed at a consistent time of day and involved an identical protocol. Data from measurement session one only, were used to address the aims of the current study. The session involved knee extensor torque and EMG measurements during MVCs and explosive voluntary contractions. TMS was used to obtain superimposed MEP and femoral nerve stimulation to obtain compound muscle action potentials at rest.

### 2.3 Torque Measurements and Surface Electromyography (EMG)

Participants were tightly secured in a custom‐built strength testing chair (fig. 6b in Maffiuletti et al. 2016) with a waist belt and shoulder straps. The hip and knee angles were set at 100° and 105°, respectively (full extension being 180°). All contractions were isometric knee extensions performed with the right leg. An ankle strap joined to a calibrated S‐shaped load cell (FSB‐1.5kN, Force Logic, Reading, UK) was secured 4 cm proximal to the medial malleolus. The force signal was amplified (×375) and then sampled at 2000 Hz (Micro3 1401 and Spike2 v.8; CED., Cambridge, UK). Offline, the force was filtered (fourth‐order low‐pass Butterworth, 250 Hz cut‐off), corrected for limb weight and multiplied by the external moment arm to calculate joint torque.

The skin was prepared by shaving, cleaning (70% ethanol) and lightly abrading the area where EMG electrodes were placed. A single, bipolar silver‐silver‐chloride gel‐electrode configuration (1.3‐cm diameter and 2‐cm inter‐electrode distance; Dual Electrode, Noraxon, Arizona, USA) was placed over the belly of each of the rectus femoris (RF), vastus lateralis (VL) and vastus medialis (VM), based on SENIAM guidelines (Hermens et al. 1999). The three wireless EMG signals (amplification ×500) were transmitted to a desktop receiver (TeleMYO D.T.S., Noraxon, Arizona, USA) and sampled, along with a single wired EMG signal (see below), at 2000 Hz via the same A/D convertor and software as the force signal. The EMG system has an inherent 312‐ms delay, so while suitable for measurements involved in the study, EMG signals sampled by it could not be used to detect activation onset and trigger the TMS in real time during the explosive contractions (explained below in experimental procedures). Thus, a second wired bipolar EMG electrode (1 cm diameter and 2 cm inter‐electrode distance; Dual Electrode, Biometrics Ltd, Gwent, UK) was placed on the belly of the VM to trigger the TMS during contraction. Electrode locations were marked with a permanent marker pen, and participants were asked to maintain these marks throughout the study by reapplying them if necessary. Offline, wireless EMG signals were filtered (fourth‐order Butterworth, band‐pass, 6–500 Hz) and time‐corrected for the 312‐ms delay inherent in the Noraxon system.

### 2.4 TMS

TMS, inducing a posterior–anterior (PA) current, with a 1‐ms pulse width was delivered via a double cone coil (110‐mm Magstim 200, Whitland, UK) over the scalp in an optimal position to elicit MEPs in the right quadriceps muscles. Participants wore a swim cap, and the vertex of the head—identified as 50% of the distance between (i) nasion and inion and (ii) right and left temporomandibular joint—was marked on the swim cap. A 5‐by‐5‐cm grid with 1‐cm spacing between grid lines was drawn on the swim cap, lateral (left hemisphere) and posterior from the vertex. A custom support was placed behind the head and neck of participants when sat in the dynamometer so they could easily maintain a comfortable head position. The coil was moved posteriorly and laterally from the vertex in ~0.5‐cm steps, and in each position, the participant completed four submaximal voluntary contractions at 20% MVC torque (established in the warm‐up; see below) with superimposed TMS on each contraction at a submaximal (range 50%–60%) stimulator output. The position which produced the highest consistent MEP amplitudes (peak‐to‐peak) over the four superimposed contractions for all three muscles (RF, VL and VM) was deemed the optimal coil position. Once established, this was marked by drawing the edge of the coil over the swim cap, which was used throughout the remainder of the session. The active motor threshold (AMT) was then determined via a series of 20% MVC torque contractions superimposed with TMS stimulator output starting at 39%. The AMT was defined as the minimum TMS intensity required to elicit five visible MEPs amongst the background EMG activity of the VM and RF, out of 10 consecutive superimposed contractions. If the muscles had less than five visible MEPs, the machine intensity was increased in steps by 2% of machine output (and conversely decreased if there were more than five visible MEPs). We prioritised the VM and RF (not the VL) in AMT decisions as these muscles produced more consistent and visible MEPs in pilot testing. Where it was impossible to match AMT for VM and RF, we settled on being one visible MEP away from 5/10 (e.g., 6/10 VM and 4/10 RF). The same investigator held the coil by hand throughout the measurement sessions, continuously monitoring its position and orientation. For TMS delivered during maximal and explosive contractions (see experimental protocol), the intensity was set at 140% of stimulator output at AMT. This intensity was chosen as it occurs on the steepest section of the input–output curve for MEP amplitude (Groppa et al. 2012; Rossini et al. 2015; Rossi et al. 2020) so it would maximise our chance of observing changes in this variable.

### 2.5 Femoral Nerve Electrical Stimulation

Single, square‐wave pulses (200 μs duration) were delivered (DS7AH, Digitimer, Hertfordshire, UK) over the femoral nerve in the inguinal triangle to evoke twitch contractions and obtain compound muscle action potentials (M‐waves) at rest. The anode (5 × 8 cm carbon rubber; EMS Physio Ltd., Oxfordshire, UK) was placed over the head of the greater trochanter. The optimal location of the cathode (1 cm diameter tip; S1 Compex Motor PointPen, Digitimer, UK) was determined as that which evoked the greatest peak twitch torque for a given submaximal stimulation intensity (80–120 mA). The cathode was taped down and held in position by the same investigator as a series of twitches at incremental intensities were evoked until there was a plateau in the peak‐to‐peak M‐wave amplitude (M_max_) of all three muscles (RF, VL and VM). The stimulator intensity was then increased to 150% of the intensity at M_max_, ensuring supramaximal intensity. Three supramaximal twitch contractions were then evoked, each separated by 15 s, and M_max_ was averaged across the three contractions for each muscle.

### 2.6 Experimental Protocol

**FIGURE 1:**
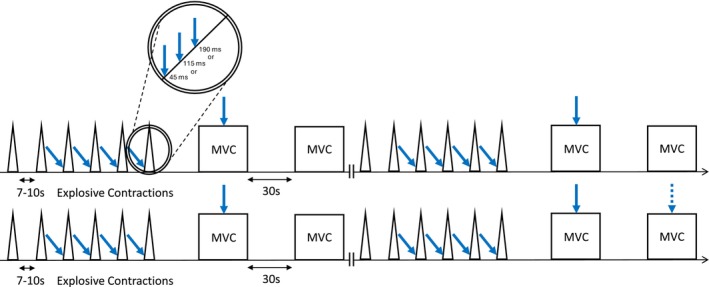
Schematic depicting a single experimental block, of which there were three during the experimental session. Each block consisted of four sets of contractions as shown. The triangles represent explosive contractions, while the squares represent MVCs. The blue arrows represent superimposed TMS applied to certain contractions. The dashed arrow above the final MVC represents a superimposed TMS applied in only one of the four sets. The blocks differed in timing of stimulation during explosive superimposed contractions, as described in the main text and depicted in the insert. For example, data refer to Figure 2.

Participants first completed a warm‐up involving a series of explosive, submaximal and maximal voluntary contractions. These MVCs were used to establish the MVC torque, defined as the highest recorded peak torque. Next, we followed the procedures described above, for obtaining optimal TMS coil position, AMT and M_max_. Participants then completed a series of MVCs and explosive voluntary contractions with and without superimposed TMS. The instruction for MVCs was to ‘push as hard as possible’ for 3–5 s and for explosive contractions to ‘push as fast and hard as possible’, emphasising fast for 1 s. The protocol was organised into three blocks of contractions. Each block (Figure 1) involved 24 explosive contractions (8 without and 16 with superimposed TMS) and 8 MVCs (3 without and 5 with superimposed TMS) distributed across four sets. Each set involved six explosive contractions (the final four with TMS stimulation) and two MVCs. In Sets 1–3, one of the two MVCs had superimposed TMS (randomly ordered), and in Set 4, both MVCs had superimposed TMS. Participants rested 10 s between explosive contractions, 30 s between MVCs, 120 s between sets and 300 s between blocks. Each block was identical except for the timing of TMS application during explosive contractions, with each block using a different TMS timing for these contractions (see next paragraph). The order of the blocks was randomised across participants. Overall, the 3‐block protocol yielded 24 explosive contractions and 9 MVCs without TMS, and 48 explosive contractions (16 per stimulus contraction phase) and 15 MVCs with TMS.

**FIGURE 2:**
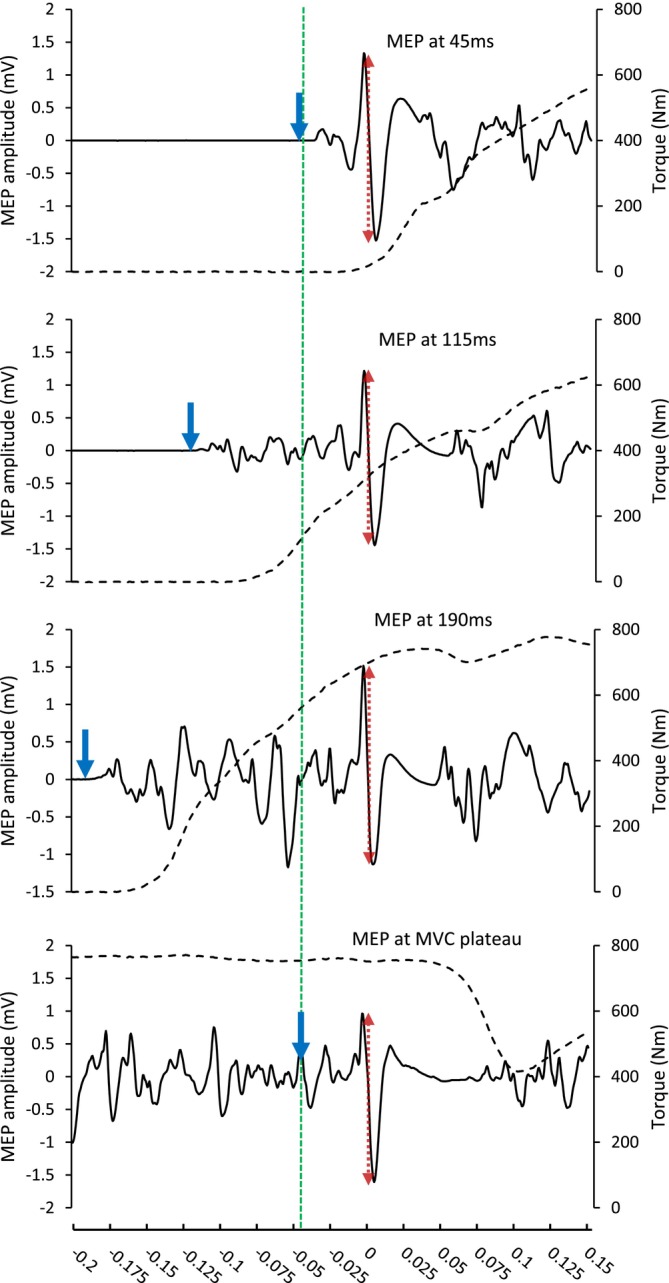
Representative traces of motor‐evoked potentials (full black line) in the vastus medialis and knee extensor torque (empty dotted line) in each of the four conditions. The downward pointing blue arrows indicate EMG on‐set for the three explosive contraction conditions (top three plots) and manual TMS trigger time for the MVC condition (bottom plot). The labels in the top three plots reflect approximate MEP times relative to EMG on‐set. The red dotted arrows represent the peak‐to‐peak amplitude of the MEPs used for analysis. EMG data are on the amplified scale (gain = 500).

For superimposed MVCs, the TMS was triggered manually by the same experienced investigator during the torque plateau of the MVC. For superimposed explosive contractions, the TMS was triggered automatically when the VM EMG signal of the wired system exceeded a set threshold. The threshold was set in the Spike2 software as the lowest amplitude for that session above the highest peaks and troughs of the baseline noise. TMS was triggered at 3, 73 or 148 ms from EMG threshold crossing for early, middle and late contraction phases, respectively. When expressed relative to manually detected EMG onset (determined via the methods of Tillin et al. 2010), TMS triggers occurred at approximately 8 (early), 78 (middle) and 153 (late) ms. Thus, our threshold was typically crossed within 5 ms of manually detected EMG onset. This TMS triggering method was based on the methods of Giboin et al. (2018). The centre of the resulting MEPs occurred at approximately 45 (early), 115 (middle) or 190 ms (late) from manually detected EMG onset (Figure 2).

### 2.7 Data Screening and Analysis

Out of 15 superimposed MVCs, only those where the TMS was delivered within 90% of MVC torque were used for further analysis. Out of the 16 superimposed explosive contractions per contraction phase, only those that met the following criteria were used for further analysis: (i) average baseline force did not change by > 2 Nm during the 200 ms preceding manually detected force onset (detected as in Tillin et al. 2010); and (ii) there was a genuine attempt at an explosive contraction. An explosive contraction with TMS was considered genuine when the highest instantaneous slope of the torque‐time curve recorded during the period prior to MEP onset, was within 3 standard deviations (SD) of the same variable recorded in the same time period, averaged across the best 3 contractions without TMS. The best 3 contractions without TMS were those with the highest peak slope (Tillin et al. 2010). Based on the above criteria, the first 10 usable contractions with TMS were used in each contraction phase for further analysis. This number was based on the following rationale: (i) our previous study (Castelli et al. 2025) showed averaging data across 10–20 contractions provided optimal reliability of the main dependent variables; (ii) the maximum number of usable contractions we could obtain in some participants in the early and middle contraction phases was 10.

For usable contractions, MEP amplitude was defined as the peak‐to‐peak of the superimposed response and is reported in absolute terms (V), normalised to M_max_ (muscle‐specific) and relative to muscle background EMG (MEP/EMG). Background EMG was RMS amplitude of the EMG in the 20 ms immediately following TMS stimulation and therefore just prior to MEP appearance, within the same contraction and muscle as the MEP. The silent period duration was measured from the stimulation point to the EMG activity resumption, after the MEP. The second derivative of EMG amplitude over time was established (1‐ms time constant) and then the signal was rectified to determine the resumption of EMG activity. EMG activity resumption was defined when the amplitude of the second derivative increased above 5 SD of the mean baseline (calculated in a 500 ms period prior to the contraction) for 70% of the next 10 ms (Figure 3 in Castelli et al. 2025). As recommended by Damron et al. (2008), the semi‐automated process was validated through manual inspection of the signal (Tillin et al. 2010; Tillin et al. 2012, 2013, 2018; Folland et al. 2014; Behrens et al. 2015; Morales‐Artacho et al. 2018; Cossich and Maffiuletti 2020). Torque was measured at discrete time points from torque onset (Maffiuletti et al. 2016), and torque onset was defined manually with a method considered the golden standard (Tillin et al. 2010). In brief, torque data were viewed on a constant x‐axis scale of 500 ms and y‐axis scale of 1 Nm, which provided good resolution from which to establish the baseline noise pattern and interpolate onset, defined as the last peak/trough before the signal deflected away from the baseline.

The median absolute deviation (MAD) process was used to detect outliers for all variables and datasets, which have been proven to be the most robust scale measure in the presence of outliers, and the most conservative value (MAD = 3) was used to define outliers. Following the process described, one participant (P14) was considered an outlier for the silent period duration, and his data were removed from further analysis involving the silent period duration. Statistical analyses, described in the following sections, were performed using R 4.4.3 (R Core Team 2025). The significance level was set at *p* ≤ 0.05.

### 2.8 Across‐Person Correlation Analysis

These analyses examined the relationships, across participants, between TMS responses (MEP normalised to M_max_ and the silent period duration) and: (i) torque for each contraction phase; and (ii) maximal rate of torque development (RTD_max_). Torque was measured at 45 (early), 115 (middle) and 190 ms (late) after torque onset in explosive contractions without TMS, normalised to MVC torque and averaged across the three contractions exhibiting the highest peak slopes (Tillin et al. 2010). The maximal rate of torque development (RTD_max_) was also measured in the best three contractions, to provide an index of rapid torque performance that is not time dependent. RTD_max_ was determined as the maximal value of the first derivative of torque, calculated using overlapping time windows from 0–1 ms up to 0–150 ms and normalised to MVT (Del Vecchio et al. 2024; Škarabot et al. 2024). Because RTD_max_ can occur over varying time windows within the first 0–150 ms, it does not consistently align with any of the time points used for TMS stimulation. RTD_max_ was therefore considered a secondary variable, with torque in each phase the primary variable, in the across‐person correlation analyses. TMS responses were averaged across the three quadriceps muscles as is normal when relating muscle activity of the quadriceps to net knee extension rapid torque (Rodriguez et al. 2022). TMS responses were also averaged across the first 10 available contractions for each contraction phase, for the reasons discussed above in ‘Data screening and analysis’. To account for muscle activation in earlier phases affecting torque in later phases (Folland et al. 2014), early and middle phase TMS responses were averaged for correlation with middle phase torque, and early, middle and late phase TMS responses were averaged for correlation to late phase torque. We were able to do this for across‐participant correlations because, unlike within‐person correlations (see below), torque and MEP data were taken from separate contractions. The data reduction yields 14 observations for the correlations involving MEP amplitude and 13 for those involving silent period duration (one per participant), which was considered insufficient to robustly assess normality. Accordingly, across‐participant correlation analyses utilised non‐parametric Spearman rho correlations. Correlations coefficients were interpreted as negligible 0 ≤ *r* ≤ 0.09, weak 0.1 ≤ *r* ≤ 0.3, moderate 0.4 ≤ *r* ≤ 0.69, strong 0.7 ≤ *r* ≤ 0.89 and very strong 0.9 ≤ *r* ≤ 1 (Schober and Schwarte 2018).

### 2.9 Within‐Person Correlation Analysis

These analyses used trial‐level data to examine the within‐person relationship between early‐phase TMS responses (absolute MEP amplitude and silent period duration) and torque measured 30 ms after torque onset within the same explosive contractions. As with between‐participant correlations, TMS responses were averaged across muscles. Middle and late phases were not assessed because correlating TMS responses with torque measured in later phases requires combining TMS responses obtained across multiple phases (see between‐group correlation analyses section) which is not possible within individual contractions. The analyses were conducted using repeated measures correlation (RmCorr), implemented via the RmCorr R package Version 0.7.0 (Bakdash and Marusich 2017), which provides an overall within‐participant correlation coefficient, and is similar to a linear mixed effects model with random intercept for participant and fixed slope. For any pair of variables, the choice of assignment between predictor and outcome variables does not affect the correlation coefficient, but it does affect the residuals of the underlying model and, thus, model evaluation. We used torque as the outcome variable as this aligns with our hypothesis that corticospinal activity is a determinant of rapid torque. In examining model residuals, we found non‐normal torque residual distributions for both analyses (both MEP amplitude and silent period duration as the predictor; Shapiro–Wilk tests, *p* < 0.01). For the reported analyses we applied square root transformation to torque, which resulted in normally distributed residuals (Shapiro–Wilk tests, *p* > 0.05). Correlation coefficients were interpreted in the same way as across‐person correlation coefficients.

### 2.10 Analysis of Contraction Phase Effects

For this analysis, TMS outcomes (absolute MEP amplitude, MEP normalised to M_max_, MEP/EMG and silent period duration) were first averaged across the first 10 available contractions for each contraction phase (see ‘Data screening and analysis’ section). Linear mixed‐effects models were then used to assess the fixed effects of Phase and Muscle and their interaction, on these outcomes. Participant was modelled as a random intercept to account for repeated measures. We used the ‘lmerTest’ R package Version 3.1‐2 to fit the models and produce Type III ANOVA tables for the fixed effects using Satterthwaite's approximation for degrees of freedom. Significant fixed effects were followed up with pairwise post hoc comparisons with Bonferroni (Leys et al. 2013) adjustment for multiple comparisons, conducted using the ‘emmeans’ R package Version 1.10.7. In examining model residuals, we found normal residual distributions for models fitted to normalised MEP and silent period duration data (Shapiro–Wilk tests, *p* > 0.05), but non‐normal residual distributions for models fitted to absolute MEP and MEP/EMG data (Shapiro–Wilk tests, *p* < 0.001). In the reported analysis, we used ln (natural logarithm) transformation of absolute MEP and MEP/EMG, which resulted in normally distributed residuals (Shapiro–Wilk tests, *p* > 0.05).

## 3 Result

Tabulated means and SDs are provided in a supplementary file, including dependent variables not presented in figures (such as muscle‐specific MEPs) and constituents of normalised variables (such as MVT, M_max_ and RMS EMG; Tables S1–S3). To check for signs of fatigue, we compared the highest MVC torque between the first set of the first block and the last set of the last block. No significant difference was found, suggesting no significant within‐session fatigue (start: 213 ± 67 Nm; end: 208 ± 64 Nm; paired *t*‐test, *p* = 0.22).

### 3.1 Across Participants Correlation

**FIGURE 3:**
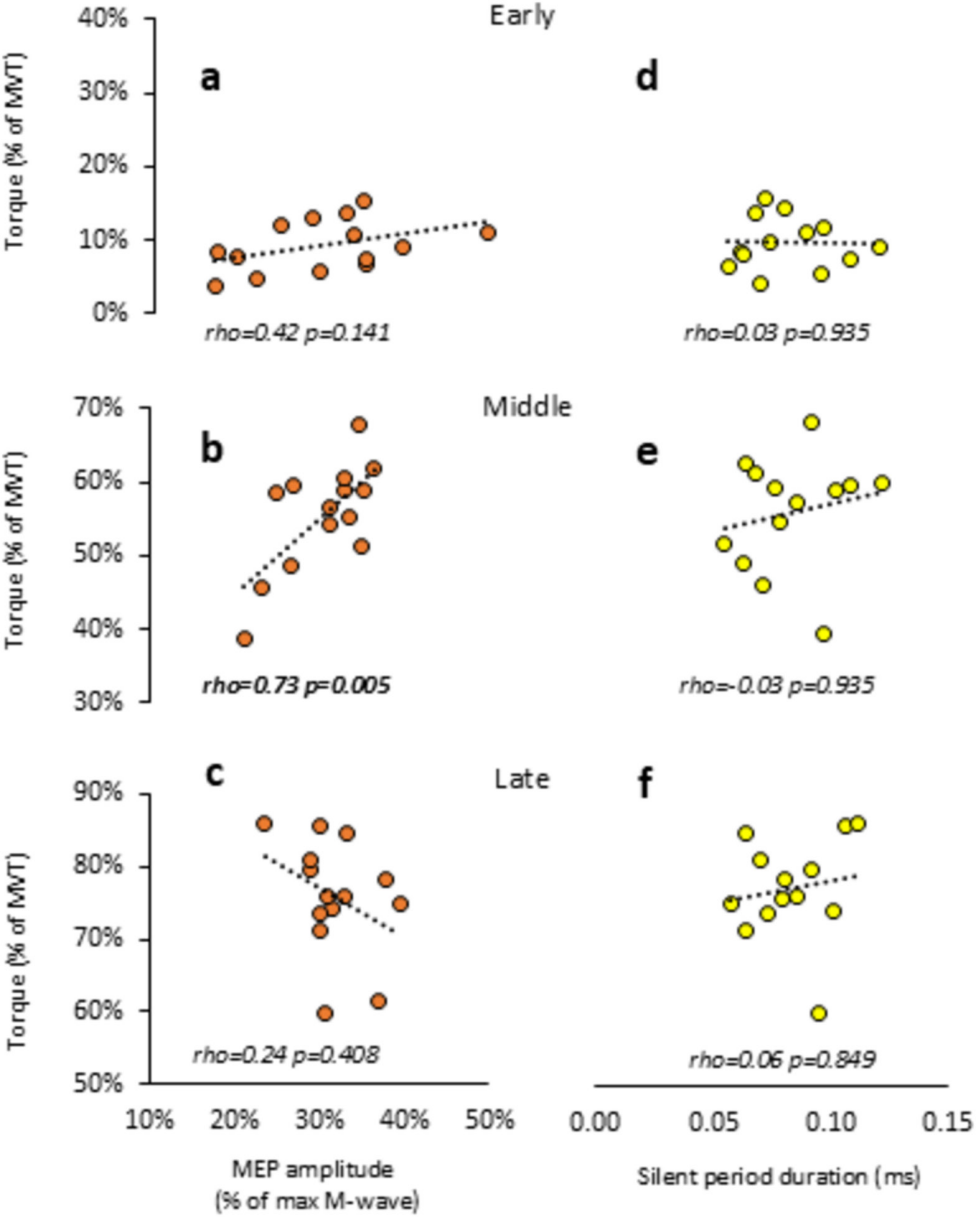
Across‐person correlations between TMS responses and normalised torque for different phases of explosive voluntary contractions. TMS responses were MEP amplitude normalised to M_max_ (a–c) and silent period duration (d, e). Explosive torque was normalised to maximal voluntary torque (MVT). The dotted lines represent linear regression lines. Spearman's rho correlation coefficient and *p*‐values are also presented. The phases of explosive voluntary contraction were early (45 ms), middle (115 ms) and late (190 ms) from EMG onset (for MEP) or torque onset (for torque). TMS responses are averaged across the three superficial quadriceps, and for the middle and late phases are an average of data from that phase and the previous phase/s. Plots (d–f) contain one less data point as silent period data was dropped for one participant.

Normalised MEP amplitude and normalised rapid torque were weakly correlated in the late phase (rho = 0.24, *p* = 0.408; Figure 3c), but moderately correlated in the early phase (rho = 0.42, *p* = 0.141; Figure 3a), and strongly correlated in the middle phase (Figure 3b) of explosive contraction, with the latter being statistically significant (rho = 0.73, *p* = 0.004). Correlations between silent period duration and normalised rapid torque were weak and non‐significant for all contraction phases (rho < 0.06, *p* > 0.849; Figure 3d–f). Normalised MEP amplitude and normalised RTD_max_ were weakly and non‐significantly correlated across all contraction phases (0.33 < rho < 0.30, *p* > 0.25). Similar results were observed for the silent period, which also showed weak and non‐significant correlations across all contraction phases (−0.09 < rho < −0.15, *p* > 0.62).

### 3.2 Within Participant Correlation

**FIGURE 4:**
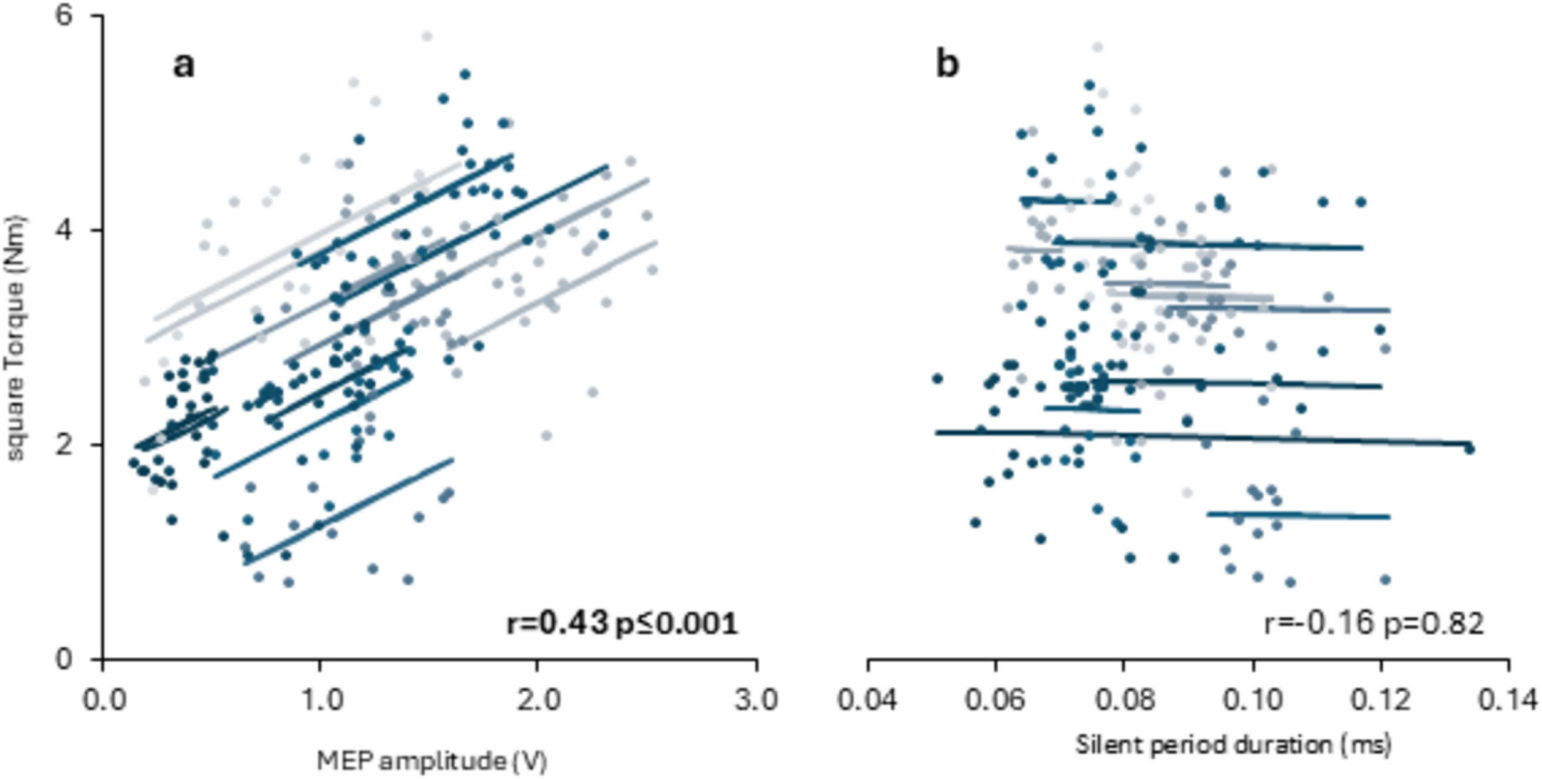
Within person (repeated measures) correlations between TMS responses and square root transformed torque in the early phase of explosive contractions. TMS responses are absolute MEP amplitude (a) and silent period duration (b). Torque was recorded at 30 ms from torque onset in the same contractions as the MEP. The dots represent individual contractions (colour‐coded by participant) and the lines represent the RmCorr fit for each participant. The correlation coefficients and *p*‐values are also provided. MEP amplitudes are on the amplified scale (gain = 500).

Repeated measures correlation using trial‐level early phase data showed a statistically moderate correlation between absolute MEP amplitude and square root transformed absolute torque (*r* = 0.43, *p* = 0.001; Figure 4a). However, early phase silent period duration was not correlated with square root transformed absolute torque (*r* = −0.02, *p* > 0.816; Figure 4b).

#### 3.2.1 Effects of Muscle and Contraction Phase

**FIGURE 5:**
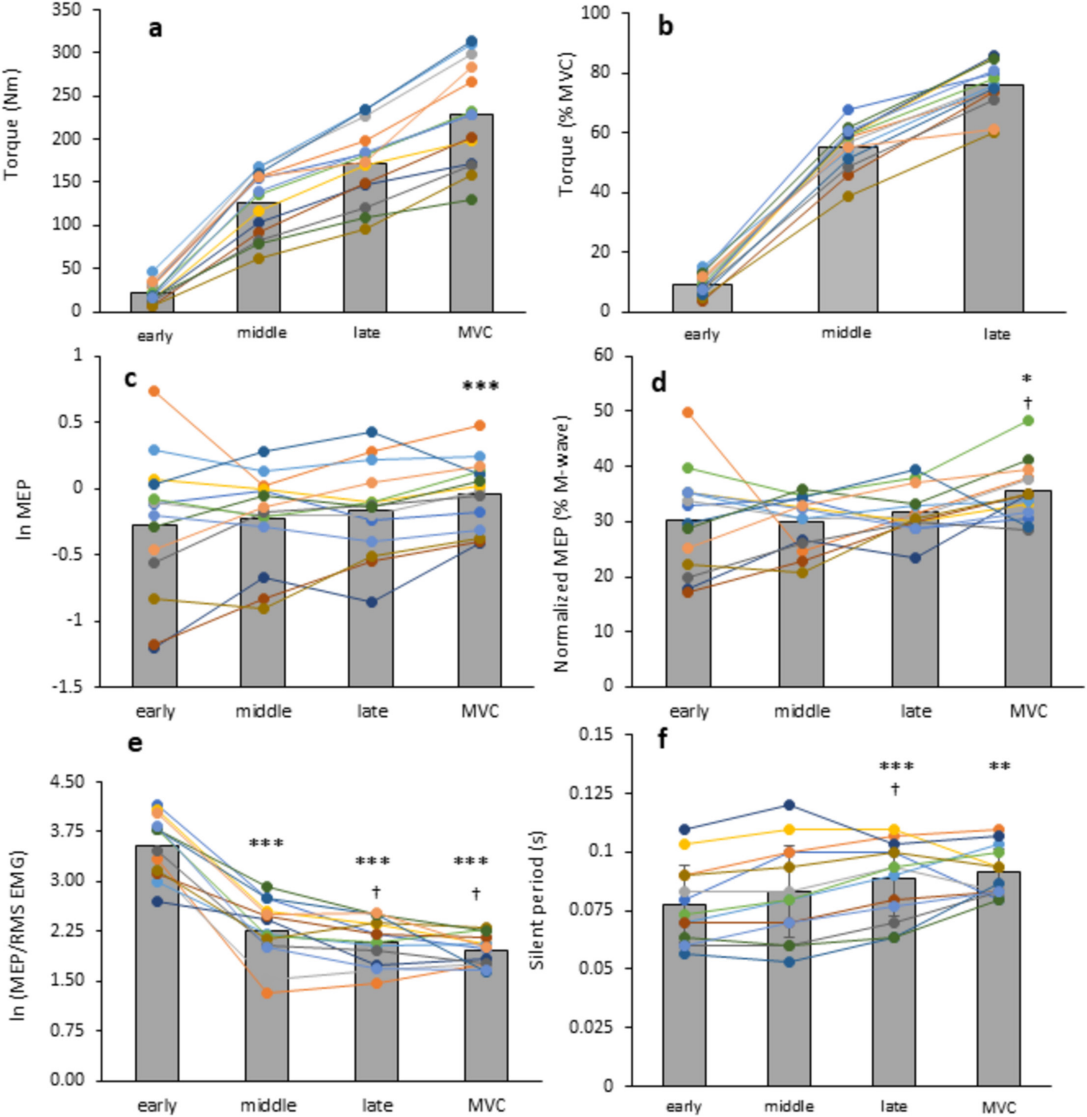
The effect of the contraction phase on absolute torque (a), torque normalised to MVT (b), absolute MEP amplitude (c), MEP amplitude normalised to M_max_ (d), MEP amplitude relative to background EMG (MEP/EMG) (e) and silent period duration (f). Background EMG is the RMS EMG recorded in the 20 ms from the TMS stimulation. Absolute MEP amplitude (c) and MEP/RMS EMG (e) were ln‐transformed prior to analysis. Prior to ln‐transformation absolute MEP amplitude was in volts on the amplified scale (gain = 500). Separate‐coloured lines and dots show data from individual participants, while the grey bars represent group averages (*n* = 14, 13 for the silent period). Data were recorded at the plateau of MVCs and during the early (45 ms), middle (115 ms) and late (190 ms) phases of explosive contractions from rest. TMS response data are averaged across the three superficial quadricep muscles. Paired differences are denoted by *** (< 0.001) or ** (< 0.001) for different to early and † (< 0.05) for different to middle.

There were significant main effects of contraction phase on all outcome variables (*p* < 0.025; Table 1; Figure 5). Post hoc pairwise comparisons (Figure 5) revealed the following: ln‐transformed absolute MEP was higher at MVC plateau than the early explosive phase (*p* = 0.021); normalised MEP was higher at MVC plateau than the early (*p* = 0.035) and middle (*p* = 0.025) explosive phases; ln‐transformed MEP/EMG was higher in the early explosive phase than all other phases (*p* < 0.001) and higher in the middle explosive phase than MVC plateau (*p* = 0.006); silent period duration was longer in both MVC plateau and the late explosive phase than in the early (*p* < 0.001 vs. both) and middle (*p* < 0.001 vs. MVC plateau; *p* = 0.017 vs. late) explosive phases. All other post hoc pairwise comparisons for phase were non‐significant (*p* > 0.05).

**TABLE 1:** *F‐* and *p‐*values for fixed effects from linear mixed effects models assessing the effects of contraction phase, muscle and their interaction (phase*muscle) on the following dependent variables: ln transformed absolute MEP amplitude, normalised MEP amplitude (normalised to M_max_), ln‐transformed MEP amplitude relative to background EMG amplitude (MEP/EMG) and silent period duration. Variables were measured across three phases of explosive contraction (early, middle and late) and at the MVC plateau in three quadriceps muscles (RF, VL and VM). Numerator degrees of freedom were 3 for Phase, 2 for Muscle, and 6 for the Phase × Muscle interaction. Denominator degrees of freedom were 143 for all models, except for the silent period where it was 132. Statistics for statistically significant effects are shown in bold.

	Absolute MEP		Normalised MEP		MEP/EMG		Silent period	
	*F*‐value	*p*‐value	*F*‐value	*p*‐value	*F*‐value	*p*‐value	*F*‐value	*p*‐value
Phase	**3.23**	**0.024**	**3.64**	**0.014**	**130.6**	**< 0.001**	**20.7**	**< 0.001**
Muscle	**91.2**	**< 0.001**	**34.0**	**< 0.001**	**10.8**	**< 0.001**	**4.51**	**0.013**
Phase*muscle	0.54	0.780	0.56	0.759	0.69	0.654	1.04	0.405

There were significant main effects of muscle on all outcome variables (*p* < 0.02; Table 1). Post hoc pairwise comparisons revealed the following: ln‐transformed absolute MEP was higher in VM than in VL (*p* < 0.001) and RF (*p* < 0.001); normalised MEP was higher in RF than in VM (*p* < 0.001) and VL (*p* < 0.001); ln‐transformed MEP/EMG was higher in VM (*p* < 0.001) and RF (*p* < 0.001) than in VL; and silent period duration was longer in RF than in VM (*p* = 0.030) and VL (*p* = 0.032). All other post hoc pairwise comparisons for muscle were non‐significant (*p* > 0.05).

There was no significant interaction between phase and muscle for any variable (Table 1).

## 4 Discussion

We observed positive correlations between MEP amplitude and rapid torque production in the early to middle phase of contraction, both between contractions within a person and across participants. This suggests corticospinal excitability may be an important determinant of rapid torque production. However, in contrast to our hypothesis, MEP amplitude was lower in the early phase of explosive contractions than at the MVC plateau, suggesting excitability is not as high as it could be at the start of an explosive contraction and that it increases throughout the rising torque‐time curve and up to MVC plateau. Consistent with our hypothesis though, the silent period duration increased with time and tension after contraction onset, being shortest in the early phase, followed by the middle and late phases, and finally the MVC plateau. These changes in silent period duration suggest corticospinal inhibition was low in the early phases but increased with torque development over time throughout an explosive contraction. Low inhibitions at the start of an explosive contraction are likely necessary for ensuring a rapid rise in torque production. Nevertheless, silent period duration was not correlated with rapid torque between contractions within a person nor across participants, which may be due to low within‐phase variability in corticospinal inhibition during an explosive contraction.

### 4.1 Association Between Torque Production and TMS Responses

This study is the first to report a correlation between MEP amplitude and rapid torque production across separate contractions within a person and across participants. These correlations were most observable for the early phase for within‐person correlation and the middle phase across participants and suggest corticospinal excitability might be an important determinant of both early‐ and middle‐phase rapid torque. It is well established that early‐ and middle‐phase rapid torque are both correlated with muscle activation (De Ruiter et al. 2007; Folland et al. 2014; Del Vecchio et al. 2019), and our results suggest greater corticospinal excitability may be required to maximise muscle activation and thus rapid torque production during an explosive contraction.

We can only speculate as to why the across‐participant correlation between MEP amplitude and torque was significant in the middle phase, but not the early phase of explosive contraction, despite previous studies reporting stronger correlations between muscle activity and torque during the early phases (Folland et al. 2014). As the middle phase MEP represents an average of both the early and middle phases, thus reflecting excitability across both phases, this averaging process likely improved the reliability of the MEP data, making it more likely to observe a correlation across participants. Additionally, rapid torque and MEP amplitude measures are more repeatable in the middle phase than in the early phase (Folland et al. 2014; Castelli et al. 2025), which would also improve the likelihood of observing a correlation in the middle phase in our study. Finally, our sample size (*n* = 14) was smaller than other studies correlating neurophysiological variables to rapid torque (*n* ≥ 20, Folland et al. 2014; Del Vecchio et al. 2019). Thus, we may have observed stronger correlations had we been able to test a greater sample size. This was unfortunately not possible because of disruptions caused by the COVID‐19 pandemic.

It is also unclear why the across‐participant correlations between RTD_max_ and MEP amplitude were not significant in any contraction phase, especially given the strong correlation previously observed between RTD_max_ and motor unit discharge rate at the start of the contraction (Del Vecchio et al. 2019; Del Vecchio et al. 2024). One issue is that RTD_max_, occurs over varying time windows across participants and contractions (range 64–148 ms). Therefore, its temporal alignment with the TMS time points is inconsistent, potentially hindering any association between the two measures.

The silent period duration was not correlated with torque or RTD_max_ in any contraction phase, either within or across participants. However, when variability is low and mean values are similar across participants, the potential for significant correlations diminishes. Using the same methods, we have previously observed low variability and similar mean values in silent period durations within and between individuals (Castelli et al. 2025), which might reduce the likelihood of detecting correlations. Nevertheless, the silent period durations we observed during the explosive contractions were shorter (78–91 ms) than those observed by others (> 100 ms) during constant load contractions and MVCs (Säisänen et al. 2008; Gruet et al. 2013). As discussed below, such low silent period durations suggest minimal corticospinal inhibition during an explosive contraction, which likely benefits rapid torque production.

### 4.2 Effects of Contraction Phase on Corticospinal Excitability and Inhibition

This is the first study to examine the influence of contraction phases during explosive contraction on MEP amplitude and silent period duration. Contrary to our hypothesis, MEP amplitude was not highest in the early phase of EMG activity. In fact, a main effect of contraction phase, independent of muscle, suggested a trend for MEP amplitude (absolute and normalised to M_max_) to increase throughout the rising torque‐time curve and up to MVC plateau. This was further supported by the significantly lower absolute MEP amplitude in the early phase compared with MVC plateau and significantly lower normalised MEP amplitude in both the early and middle phases compared with MVC plateau. This contrasts with the observations of Mackinnon and Rothwell (2000) for dynamic actions, where MEP amplitude was observed to increase just prior to EMG onset, peak in the early phase of agonist activity, just prior to movement onset, then decrease during the subsequent movement. However, these dynamic rapid movements were characterised by a triphasic agonist–antagonist–agonist activation pattern, with agonist corticospinal excitability first increasing, then decreasing for the antagonist phase and then increasing again during the second agonist phase (Mackinnon and Rothwell 2000). In the present study, while we did not measure MEP amplitude in the lead up to EMG onset, we speculate that corticospinal excitability increases just prior to muscle activity, as has been shown by Baudry and Duchateau (2021) for isometric explosive contraction. Assuming this was the case in our study, we propose an increase in corticospinal excitability continues into the early and middle phase but plateaus during the late phase of explosive contraction, at a level equivalent to that observed Tillin et al. (2018) during MVC. Future research should explore this hypothesis.

The MEP/EMG was considerably greater in the early phase compared with all other phases and greater in the middle phase compared with MVC plateau (Figure 5a). Thus, corticospinal excitability is disproportionate with muscle activation in the early to middle phases of contraction but is in proportion to muscle activation at later phases and at the MVC plateau. Similar effects have been observed during rapid dynamic wrist movements by Mackinnon and Rothwell (2000), who suggested the greater MEP/EMG in the early phase of EMG activity was due to higher subthreshold excitability at a cortical level, preceding spinal and then muscle activation. The authors ruled out the increases in MEP/EMG being caused by changes at a spinal level because the H‐wave/EMG ratio remained constant throughout all contraction phases. We therefore speculate that cortical excitability increases ahead of muscle activation at the start of an explosive contraction, and it takes until the late phase for muscle activation to ‘catch‐up’ and is in proportion with cortical excitability thereafter.

Our results provide novel evidence that the silent period duration generally increased throughout an explosive contraction and up to MVC plateau, with it being lower in the early phase than in the late phase and MVC plateau, and lower in the middle phase than in the late phase (Figure 5f). Shorter silent period durations in the early phase support the notion that inhibition at a spinal and supraspinal level is low to maximise muscle activation and rapid torque development during an explosive contraction. However, corticospinal inhibition appears to increase with increased torque development during the explosive contraction and is highest at MVC plateau. The mechanisms underpinning this effect are unclear, but the silent period durations may provide some clues (Škarabot et al. 2019). The short silent period durations we observed during the different phases of explosive contraction (78–90 ms) indicate that inhibitions at a spinal level (rather than supraspinal) are driving variability in the silent period. Contributing factors to these spinal inhibitions are likely to include some motor units being in a hyperpolarised state at the time of TMS, Renshaw cell inhibition, reciprocal inhibition and/or di‐synaptic inhibition (Triggs et al. 1993; Ziemann et al. 1993; Enoka and Fuglevand 2001); all caused by activation of the motor units and/or torque development in the early phase of contraction. In contrast, the silent period durations towards the later phase of explosive contraction (86 ms) and at MVC plateau (92 ms) indicate that cortical mechanisms—likely involving intracortical inhibition due to GABA B receptor activity (in addition to spinal inhibitions)—are contributing to silent period length (Werhahn et al. 1999; McDonnell et al. 2006). The lower corticospinal inhibitions in the early phase of contraction followed by increased inhibition at later phases and at MVC plateau, potentially explain previous observations of muscle activation being +20% greater in early to middle phases of explosive contraction compared with late phase and MVC plateau (Tillin et al. 2018).

### 4.3 Limitations

It is possible MEP amplitudes measured in our study did not change proportionally with corticospinal excitability, which could mask possible differences between the phases. Evidence for this is based on studies showing that MEP amplitudes increase with increases in constant force output up to 55%–75% MVC and then plateau or slightly decrease beyond this intensity (Todd et al. 2003; Goodall et al. 2009; Škarabot et al. 2018). This is likely attributable to the high motoneuron discharge rates observed during maximal effort (explosive and MVC) contractions. Under these conditions, there is an increased likelihood that many motor units will be in their refractory phase, reducing the probability of them responding to TMS. Nevertheless, we did observe effects of contraction phase on MEP amplitude and correlations between MEP amplitude and rapid torque, suggesting the MEPs were not saturated, and variability was sufficient for addressing the aims of our study.

Explosive contractions performed in our study were from a resting state only. Therefore, we cannot be certain how our results might differ for explosive contractions performed from a state of active tension. This should be explored in future research given that rapid torque production and the motor unit activity driving it are influenced by contractile history. We cannot empirically establish the specific site (e.g., cortical, intracortical or spinal) of excitation and inhibition in the different contraction phases with TMS alone. We recommend that future studies use additional approaches (e.g., spinal stimulation procedures or methods that capture subthreshold rise in corticospinal excitability as for Mackinnon and Rothwell 2000) to explore our observations further.

## 5 Conclusion

MEP amplitude was correlated with rapid torque in the early phase (within participants) and middle phase (across participants) of explosive contraction, suggesting corticospinal excitability is an important determinant of rapid torque. MEP amplitude increased during the rising torque‐time curve, before plateauing at a level equivalent to MVC plateau. This coincided with greater MEP/EMG in the early and middle phases compared with later phases and MVC plateau. This suggests a rise in corticospinal excitability during the early to middle phases of an explosive contraction is subthreshold for muscle activation, which catches up in later phases of torque output. The silent period duration was lowest in the early phase and increased throughout the different phases, suggesting that corticospinal inhibition increases with increased torque during an explosive contraction and appears highest at the MVC plateau. Low corticospinal inhibition in the early phases of an explosive contraction maybe necessary for maximising rapid torque output.

## Author Contributions

Conception and design of the work: F. C., N. A. T., A. B. and O. S. M. Acquisition: F. C. and A. C. V. Analysis or interpretation of data for the work: F. C., N. A. T., A. B., O. S. M. and R. H. Drafting the work: F. C., N. A. T. and O. S. M. Revising it critically for important intellectual content: F. C., N. A. T., A. B., O. S. M. and R. H. All authors have read and approved the final version of this manuscript and agree to be accountable for all aspects of the work in ensuring that questions related to the accuracy or integrity of any part of the work are appropriately investigated and resolved. All persons designated as authors qualify for authorship, and all those who qualify for authorship are listed.

## Conflicts of Interest

The authors declare no conflicts of interest.

## Supporting information


**Table S1:** Descriptive data (mean ± SD) for the torque dependent variables. Units are Nm.
**Table S2:** Descriptive data (mean ± SD) for the EMG RMS amplitude dependent variables in the vastus medialis (VM), vastus lateralis (VL), rectus femoris (RF), and mean quadriceps value (Quads). Units are V and presented on amplified scale (gain = 500).
**Table S3:** Descriptive data (mean ± SD) for the MEP amplitude, silent period durations, and maximum M‐wave amplitude in the vastus medialis (VM), vastus lateralis (VL), rectus femoris (RF), and mean quadriceps value (Quads). For MEP and M‐wave, units are V and presented on amplified scale (gain = 500). Units are s for silent period.

## Data Availability

The unidentified data are available from the first author upon request.
