# Supplementary material for: Corticospinal Excitability During Explosive Voluntary Contractions and Its Association With Rapid Torque Production
Source: Eur J Neurosci. 2025 Nov 18;62(10):e70321. doi: 10.1111/ejn.70321 (PMC12627274; doi:10.1111/ejn.70321)
Supplement: Supplementary file 1 — Table S1: Descriptive data (mean ± SD) for the torque dependent variables. Units are Nm. Table S2: Descriptive data (mean ± SD) for the EMG RMS amplitude dependent variables in the vastus medialis (VM), vastus lateralis (VL), rectus femoris (RF), and mean quadriceps value (Quads). Units are V and presented on amplified scale (gain = 500). Table S3: Descriptive data (mean ± SD) for the MEP amplitude, silent period durations, and maximum M‐wave amplitude in the vastus medialis (VM), vastus lateralis (VL), rectus femoris (RF), and mean quadriceps value (Quads). For MEP and M‐wave, units are V and presented on amplified scale (gain = 500). Units are s for silent period. [file EJN-62-0-s001.pdf]

Supplementary File

Table S1. Descriptive data (mean ± SD) for the torque dependent variables. Units are Nm

	Mean ± SD
MVT	227 ± 56
Early-phase without TMS	22 ± 13
Middle-phase without TMS	131 ± 40
Late-phase without TMS	176 ± 50
Early-phase with TMS	8 ± 6
RTD_{max} (*Nm/s*)	1247.6 ± 459.8

Table S2. Descriptive data (mean ± SD) for the EMG RMS amplitude dependent variables in the vastus medialis (VM), vastus lateralis (VL), rectus femoris (RF), and mean quadriceps value (Quads). Units are V and presented on amplified scale (gain = 500).

	VM<br>Mean ± SD	VL<br>Mean ± SD	RF<br>Mean ± SD	Quads<br>Mean ± SD
MVC plateau	0.443 ±0.188	0.284 ± 0.188	0.218 ± 0.082	0.315 ± 0.167
Early-phase without TMS	0.108 ± 0.081	0.065 ± 0.058	0.050 ± 0.034	0.074 ± 0.065
Middle-phase without TMS	0.383 ± 0.211	0.206 ± 0.132	0.147 ± 0.084	0.246 ± 0.182
Late-phase without TMS	0.419 ± 0.170	0.244 ± 0.154	0.154 ± 0.086	0.272 ± 0.179
Early-phase with TMS	0.114 ± 0.085	0.069 ± 0.061	0.052 ± 0.035	0.078 ± 0.069

Table S3. Descriptive data (mean ± SD) for the MEP amplitude, silent period durations, and maximum M-wave amplitude in the vastus medialis (VM), vastus lateralis (VL), rectus femoris (RF), and mean quadriceps value (Quads). For MEP and M-wave, units are V and presented on amplified scale (gain = 500). Units are s for silent period.

	VM<br>Mean ± SD	VL<br>Mean ± SD	RF<br>Mean ± SD	Quads<br>Mean ± SD
MEP at MVC plateau	1.70 ± 0.71	0.86 ± 0.35	0.75 ± 0.21	1.10 ± 0.31
MEP at early-phase	1.46 ± 0.70	0.73 ± 0.55	0.71 ± 0.33	0.96 ± 0.27
MEP at middle-phase	1.58 ± 0.53	0.67 ± 0.34	0.65 ± 0.23	0.97 ± 0.31
MEP at late-phase	1.56 ± 0.53	0.73 ± 0.44	0.73 ± 0.28	1.01 ± 0.31
Silent period at MVC plateau	0.088 ± 0.011	0.087 ± 0.010	0.097 ± 0.009	0.092 ± 0.009
Silent period at early-phase	0.077 ± 0.016	0.078 ± 0.017	0.080 ± 0.018	0.078 ± 0.013
Silent period at middle-phase	0.082 ± 0.019	0.083 ± 0.019	0.084 ± 0.019	0.083 ± 0.016
Silent period at late-phase	0.087 ± 0.016	0.087 ± 0.016	0.091 ± 0.016	0.087 ± 0.014
Maximal M-wave	5.3 ± 1.4	2.9 ± 1.5	1.8 ± 0.4	N.A.
